# Intra- and Extra-Hospitalization Monitoring of Vital Signs—Two Sides of the Same Coin: Perspectives from LIMS and Greenline-HT Study Operators

**DOI:** 10.3390/s23125408

**Published:** 2023-06-07

**Authors:** Filomena Pietrantonio, Antonio Vinci, Massimo Maurici, Tiziana Ciarambino, Barbara Galli, Alessandro Signorini, Vincenzo Mirco La Fazia, Francescantonio Rosselli, Luca Fortunato, Rosa Iodice, Marco Materazzo, Alessandro Ciuca, Lamberto Carlo Maria Cicerchia, Matteo Ruggeri, Dario Manfellotto, Francesco Rosiello, Andrea Moriconi

**Affiliations:** 1 Internal Medicine Unit, Castelli Hospital, Asl Roma 6, 00072 Rome, Italy; 2 Departmental Faculty of Medicine, St. Camillus University of Medicine and Health Sciences, 00131 Rome, Italy; alessandro.signorini@unicamillus.org (A.S.); mruggeri76@gmail.com (M.R.); 3 Local Health Authority Roma 1, 00193 Rome, Italy; antonio.vinci.at@hotmail.it; 4 Doctoral School in Nursing Science and Public Health, University of Rome “Tor Vergata”, 00133 Rome, Italy; 5 Department of Biomedicine and Prevention, University of Rome “Tor Vergata”, 00133 Rome, Italy; maurici@med.uniroma2.it; 6 Department of Internal Medicine, Luigi Vanvitelli University, 81100 Caserta, Italy; tiziana.ciarambino@gmail.com; 7 Casa Circondariale Rebibbia, Nuovo Complesso Prison, 00185 Rome, Italy; barbaragallimed@gmail.com; 8 Texas Cardiac Arrhythmia Institute, St. David’s Medical Center, Austin, TX 78705, USA; vmirco.lafazia@gmail.com; 9 Department of Systems Medicine, Division of Cardiology, Tor Vergata University, 00133 Rome, Italy; 10 Cardiology and Coronary Intensive Therapy Unit, San Francesco di Paola Hospital, 87027 Paola, Italy; rossellifrancesco@alice.it; 11 Studio Legale Fortunato, 00100 Roma, Italy; lucafortunato.85@gmail.com (L.F.);; 12 Breast Unit, Department of Surgical Science, Policlinico Tor Vergata University, 00100 Rome, Italy; marco.materazzo@alumni.uniroma2.eu; 13 PhD Program in Applied Medical-Surgical Sciences, Breast Oncoplastic Surgery, University of Rome Tor Vergata, 00100 Rome, Italy; 14 Department of Infectious Disease and Public Health, Sapienza University of Rome, 00185 Roma, Italy; ciuca.alessandro@gmail.com; 15 General practitioner School, ASL Latina, 01100 Latina, Italy; studiomedicocicerchialamberto@gmail.com; 16 Local Health Authority Roma 5, 00036 Palestrina, Italy; 17 National Centre for Health Technology Assessment, National Health Institute, 00153 Rome, Italy; 18 UOC Medicina Interna, Fatebenefratelli Isola Tiberina-Gemelli Hospital, 00186 Rome, Italy; dario.manfellotto@afar.it; 19 “Centro Studi” FADOI, 20123 Milan, Italy; 20 Department of Hystological, Anatomical Sciences and Legal Medicine, Sapienza-University of Rome, 00196 Rome, Italy; 21 Department of Business and Management, LUISS University, 00197 Rome, Italy; andrea.moriconi98@gmail.com

**Keywords:** telemedicine, healthcare management, staff satisfaction, quality in healthcare, doctor–patient relationship, future vision

## Abstract

Background: In recent years, due to the epidemiological transition, the burden of very complex patients in hospital wards has increased. Telemedicine usage appears to be a potential high-impact factor in helping with patient management, allowing hospital personnel to assess conditions in out-of-hospital scenarios. Methods: To investigate the management of chronic patients during both hospitalization for disease and discharge, randomized studies (LIMS and Greenline-HT) are ongoing in the Internal Medicine Unit at ASL Roma 6 Castelli Hospital. The study endpoints are clinical outcomes (from a patient’s perspective). In this perspective paper, the main findings of these studies, from the operators’ point of view, are reported. Operator opinions were collected from structured and unstructured surveys conducted among the staff involved, and their main themes are reported in a narrative manner. Results: Telemonitoring appears to be linked to a reduction in side-events and side-effects, which represent some of most commons risk factors for re-hospitalization and for delayed discharge during hospitalization. The main perceived advantages are increased patient safety and the quick response in case of emergency. The main disadvantages are believed to be related to low patient compliance and an infrastructural lack of optimization. Conclusions: The evidence of wireless monitoring studies, combined with the analysis of activity data, suggests the need for a model of patient management that envisages an increase in the territory of structures capable of offering patients subacute care (the possibility of antibiotic treatments, blood transfusions, infusion support, and pain therapy) for the timely management of chronic patients in the terminal phase, for which treatment in acute wards must be guaranteed only for a limited time for the management of the acute phase of their diseases.

## 1. Introduction

### 1.1. Telemedicine and Its Potential Applications in Italy

Telemedicine encompasses a set of medical practices that involve the usage of digital technologies, with the aim of providing patients residing outside standard health structures with the healthcare they need [1,2,3]. Since telemedicine solely requires access to a virtual telecommunication channel, it stands out as being the simplest practice within the discipline of digital health [4,5,6].

Telemedicine can be categorized, on one hand, by implemented activities (i.e., televisits, teleconsultations, and tele-healthcare cooperation), and on the other, by activity purposes, which can be summarized as follows:

- Remote monitoring: A variety of medical-health activities with the purpose of monitoring a patient’s health status, via the implementation of routine medical tests, the communication of medical results to healthcare professionals, and the potential transmission of automated responses [7,8,9].
- The collection and sharing of clinical data, which aims to disseminate clinical information that is less sensitive to time between healthcare professionals and/or between doctors and patients, and which currently often involves a delay between the transmission, the receipt of, and the response to the content that was shared [10,11].
- Interactive synchronous telemedicine, which includes practices involving real-time communication between doctors and patients, which may or may not involve the activity of data sharing [12,13].

Due to the relevance of digital health tools and the benefits derived from them, a significant increase in terms of their diffusion has been observed [14]. They are included in the vast field of digital health, which comprises, among others, digital wellbeing (e.g., the use of digital applications for well-being and prevention), mobile health, medical robotics, digital therapies, and advanced diagnostics assisted by artificial intelligence, algorithms, and big data [15]. The optimization of medical prescription, such as antibiotic therapy, has also been reported [16,17].

The practical applications and digital health techniques that have emerged recently are numerous, and continue to represent a concrete opportunity to address the critical issues in health systems, offering a wide range of possibilities:

- Decreasing the timing of certain clinical therapies and treatments [18];
- Enhancing the coverage in terms of healthcare information, with a particular emphasis on information regarding the methods of prevention of various medical pathologies [19];
- Improving the coverage and quality of health services [20];
- Increasing the level of accuracy in diagnoses and the degree of precision in medical procedures [21];
- Increasing the efficiency, effectiveness, and sustainability of healthcare tools [22];
- Italian territories present a profound absence of digitalization, and a peculiar dispersion of population and services among small towns and large cities, with several instances of service availability imbalance [23].

In this scenario, the design of practices, models, and methods capable of encouraging the effective and widespread adoption of digital technologies in the healthcare sector would allow hospitals to provide services that are more in line with the expectations and needs of the population, as well as to improve the relationship between the quality of the services offered and their costs [24]. In turn, this would limit the expenditure of pecuniary and non-pecuniary resources that is generated by eventual structural inefficiencies [25].

On the other hand, several aspects of telemedicine implementation have been subject to debate and critique. For instance, it is believed to have limited utility as a standalone clinical service, with cultural, structural, and skill barriers seen as the main obstacles to its full implementation [26]. Other important barriers are technical incompatibilities, patients’ clinical conditions, such as hearing, vision, or cognitive impairment, and even a lack of seriousness from the patient. Cultural resistance is also mentioned by many sources [27].

In Italy, despite promising experience, the widespread diffusion of telemedicine services has not yet occurred, for a wide range of reasons mostly pertaining to structural networks, a lack of legislative coverage, or cultural attitudes [28,29]. Currently, a lot of evidence is collected on elderly/rehabilitation patients, while telemedicine’s benefits for the pediatric population are still matter of debate [30,31]. On the other hand, while many studies in Italy are concerned with patient outcomes and cost/benefit impact, there is little evidence on the caregivers’ side, especially concerning ease and willingness of use.

### 1.2. Objectives

With this paper, we aim to provide operators insights into two main points:

1. We question the effect of an implemented monitoring system inside a healthcare structure: How can technology adoption improve the management of patients inside a healthcare structure?
2. We examine how the implementation of technological and digital tools can help with an out-of-hospital setting change: How can health monitoring devices support the healing path of discharged patients at home?

## 2. Materials and Methods

### 2.1. Main Design

This paper investigates the qualitative aspect of telemedicine operators via a survey of a purposive sample comprising the involved operators [32]. The preliminary findings of two ongoing Italian studies (LIMS: LIght Monitoring Study and Greenline-HT: Greenline Hospital-Territory Study) are discussed while considering the involved staff’s opinions [14,33]. The choice of sampling operators from these two studies was based on opportunity and the availability of their operators, who volunteered to participate in a qualitative survey on their own ongoing trials.

“ASL Roma 6”, where the qualitative research on telemedicine was conducted, is a typical Italian Local Healthcare Facility (Azienda Sanitaria Locale, ASL) and is the administrative, commissioning, and service provision center for everything related to public healthcare under the National Healthcare Service in Italy. It is staffed by 3312 employees (including administration and healthcare providers). It includes 4 hospital structures, 8 medium-intensity private structures, and at least 36 low-intensity territorial facilities for vulnerable patients [20,34].

### 2.2. Primary Study Design

Both clinical trials were performed using a continuous vital signs monitoring system (WIN@Hospital system, Ab medica, Italy). LIMS data were collected by the Internal Medicine Unit of Manerbio Hospital (ASST-Garda) and ASL Roma 6, while Greenline-HT data were collected by ASL Roma 6. The Medical Statistics Unit of the University of Modena and Reggio Emilia UNIMORE, Modena, Italy, participated in the outcome analysis.

- LIMS Study

The LIMS study investigates the application of telemedicine by “ASST–Garda” and ASL Roma 6. It is a randomized controlled study, where patients are subjected to two different monitoring systems (traditional vs. wireless), in a random manner, such that different prognostic factors that may affect the outcome of monitoring are equally distributed in the groups that are being compared.

A qualitative survey has been conducted by the very same researchers, with the aim of extrapolating the impressions gained and the impact on management practices.

- Greenline-HT Study

The Greenline-HT study is an open-label randomized clinical trial conducted inside the Internal Medicine Wards of Castelli Hospital, Lazio. This study is organized by The FADOI Foundation (Italian Scientific Society of Internal Medicine), with the collaborative support of ASL (Local Health Authority) Roma 6. It examines the efficacy of the continuous wireless monitoring of vital parameters in reducing prevalence of major complications and in improving the clinical outcomes and quality of care of patients discharged at home.

A total of 110 patients (56 M, 54 F; mean age: 76.2) were enrolled in the Greenline-HT Study.

The possibility of gathering valuable data on the management of patients using innovative digital devices allowed us to assess the real impact of digital medicine in the healthcare organization.

### 2.3. Staff Survey and Interviews: Participants and Design

#### 2.3.1. LIMS Study

The survey was targeted at medical staff who operated the new wireless monitoring system. The survey was carried out by 16 operators. The internal survey consisted of eight different questions to assess the level of satisfaction with the introduction of software-based management and electronic medical devices for patient care.

The gender of the survey participants was omitted because it was not essential to the research, while the age and years of experience in the sector were included to obtain an overview of the participants’ characteristics. The questionnaire was administered through the Qualtrics platform, enabling participants to submit their impressions both voluntarily and anonymously.

The questions asked during the survey are presented in Table 1.

#### 2.3.2. Greenline-HTStudy

Four researchers who participated in the Greenline- HTStudy were subject to an unstructured peer interview, which aimed to let major themes emerge in a friendly environment. In line with the study’s scope, the interview was conducted online, using a common web-meeting platform.

## 3. Results

### 3.1. LIMS Study: Patient Outcomes

The LIMS study showed that telemonitoring through digital health devices leads to better patient management. The experimental arm (wireless monitored patients) and control arm behaved very differently: the occurrence of major complications dropped from 44% to 30% among the monitored patients; the mortality rate decreased from 16 to 9 percent; and there was an increase in the preventive detection of diseases, especially arrhythmia (1.9% to 4.3%) and respiratory failure (3.8% to 6.5%). In the experimental arm, nurses reduced the amount of time spent monitoring each patient each day by at least 49.6 min to a maximum of 58.1 min, which may have resulted in more time being spent on other tasks that improve patient welfare. Additionally, a 4% reduction in the readmission of patients 21 days after discharge was observed [33].

### 3.2. LIMS Study Survey

The main findings of the internal survey are based on opinions expressed about the change in the management strategy for wireless monitoring adoption. The results are summarized in Table 2.

### 3.3. Greenline-HT Study Patient Outcomes

The remote monitoring of the health status of patients through the collection and sharing of clinical data gathered by the monitoring device achieved better outcomes in patients discharged at home compared to those who were not monitored.

The lower incidence of major complications, and consequently, of re-hospitalizations in the wireless arm is a demonstration of the effectiveness of the telematic network between healthcare personnel and patients. From a management perspective, the results highlight the opportunities offered by the wireless monitoring systems: the improvement in the healing process of patients and the reduction in costs associated with hospitalizations.

The use of telemonitoring after discharge reduced the re-hospitalization event rate from day 5 onward. The largest difference was seen for events occurring from day 10 onward, although the difference was not statistically significant (*p* = 0.243) [14].

### 3.4. Greenline-HT Study Interviews

The unstructured interview involved four clinicians, (two male and two female) aged 30–37 who provided direct care to inpatients. They interviewed each other and contextually noted the main themes of the answers.

All the clinicians involved in the study were supportive of telemedicine’s involvement in day-to-day clinical practice. They were satisfied with the system and did not have remarks on clinical management. The main satisfaction theme that emerged was related to the possibility of detecting early warning signs, and to be able to adjust home therapy after discharge, reducing the chances of re-hospitalization. Most concerns were related to infrastructure problematics (low bandwidth, occasional video/audio disturbances) that are related to the technical infrastructure rather than to the system itself. Another point of concern was the ability to correctly communicate the importance of telemonitoring to the patients and their homecare providers, ensuring their compliance and minimizing hazards due to voluntary or involuntary disconnection.

## 4. Discussion

The results of our surveys confirm the findings from the literature, i.e., implementing a digital health system in patient care increases the level of accuracy in diagnoses and the overall quality of health services. In the described situations, health care workers positively embraced the integration of electronic health devices in patient management, despite the difficulties of using a new system for patient care and the need to develop digital competencies.

The main advantages and disadvantages perceived in the implementation of wireless monitoring devices are summarized via a SWOT matrix in Table 3 [35]. Increased effectiveness and improved patient safety were the two positive effects that were most frequently reported among the interviewees. The main advantages were represented by the increased safety for the patient and by the quick response in case of emergency. The major disadvantages, on the other hand, pertained to the risks involved when patients are not compliant.

The Greenline-HT results add evidence to the notion that the telemonitoring of patients discharged at home can improve their recovery process. Additionally, the lower recurrence of major complications may be the main factor that led to the recording of fewer re-hospitalizations in the monitored patients. Although the result did not achieve statistical significance, probably due to the need for the full sample size to be reached, it represents a significant starting point for further research that may fill the gaps and limitations present in the study.

## 5. Conclusions

Healthcare management modernization via telemedicine shows encouraging preliminary results. The implementation of medical monitoring devices allows for increased safety and quality of care and reduced costs. Checking patient vital signs is an essential function of patient management; however, given that it is a simple and repetitive task, it can be accomplished using technological and digital tools [36,37,38]. Personnel efficiency would thus be maximized by using human staff for essential assignments. The benefits gained from the proper allocation of human resources may contribute to alleviating the shortage of workforce that characterizes the field [39,40,41,42].

We believe that the raised questions on digital health can be answered as follows:

1. How can technology adoption improve the management of patients inside a healthcare structure?
  ○ Telemedicine guarantees the existence of a real-time information network that is permanently accessible to care providers, supporting health therapies and allowing for a constant relationship between patients and therapists. Additionally, it allows for a more rapid and natural information sharing process among different figures involved in the assistance process.
2. How can health monitoring devices support the healing path of discharged patients at home?
  ○ Patient discharge represents a crucial point in the healing pathway because it is essential to correctly draft the discharge summary, to provide extensive information to the patient and family members, and to prescribe a suitable treatment to maximize response rates. Health monitoring devices allow for the optimization and automatization of the monitoring process, allowing discharged patients to be monitored for early signs of worsening condition, and potentially preventing re-hospitalization.

Telemedicine can be used for prevention purposes, especially secondary and tertiary prevention concerning people already classified as being at risk, and people who are already ill, who must undergo constant monitoring of certain vital parameters to reduce the risk of the onset of complications.

Other opportunities that can be generated by telemedicine services are the definition of a health system that is characterized by equity and equal access to assistance and treatment, regardless of the demographic, socio-economic, and geographical characteristics of an individual [43].

Furthermore, because there is no longer the necessity to physically move from one place to another to undergo therapies, telemedicine implies a reduction in administration times, therefore diminishing the expenses for both the patient and the government. This allows for an optimal and futuristic model of healthcare, in which medical actors have the possibility to establish a personal bond with patients through multiple daily visits, taking full responsibility for the critical health conditions with which they were required to intervene [44]. This is extremely important in the management of chronic conditions, which often involves the inappropriate usage of emergency services for pathologies that, if well managed, could be treated in settings outside of the hospital [45].

For instance, during the COVID-19 pandemic, despite the overall preparedness of the healthcare service, criticalities emerged concerning the insufficiency of healthcare structures to accommodate patients who, ready for discharge from hospital, were not yet in an appropriate condition to treat themselves independently at home [46]. This lead both to an abnormal use of emergency services and inpatient wards bottlenecking [47,48,49,50,51].

Widening the wireless monitoring system to domestic settings, and hopefully to intermediate care setting, may guarantee expert assistance after hospital discharge due to the hospital’s direct network connection; moreover, it may reduce overcrowding in emergency departments and hospital wards, thanks to the preemptive role of remote-supported diagnosis and remote-aided local intervention. This was already proven an effective strategy during the COVID-19 pandemic [52].

## Figures and Tables

**Table 1:** LIMS study survey questions.

QUESTIONS	ANSWERS
What is your age?	(1) 20–30 (2) 31–40 (3) 41–50 (4) More than 50
How many years have you been working in the Healthcare field?	(1) 1–5 yrs. (2) 6–10 yrs. (3) 11–15 yrs. (4) 16–20 yrs. (5) More than 20 yrs.
Does the use of wireless monitoring device allow you to be more effective and efficient in the care of patients?	(1) Very little (1–3 pts) (2) A little (3–5 pts) (3) Fairly (5–7 pts) (4) Very much (8–10 pts)
Has it been able to establish a bond with the patient through digital visits as well?	(1) Less compared to the traditional face to face visit (2) No difference with the traditional visit (3) Slightly better than a traditional visit (4) Definitely better than a traditional visit
In your opinion, is the device easy to use? (system activation, application time, routine use, battery charging, etc.).	(1) It’s very difficult to use (2) After a short practice the device is easy to use (3) The device is very intuitive and easy to use
Have you noticed a significant reduction in the time taken to detect vital parameters?	(1) No reduction observed (2) The reduction is minimal (3) A slight reduction in the time taken to ditect vital parameters (4) A significant reduction in the time taken to detect vital parameters
Since the start of the project, have you noticed any changes in qualitative point of view, both in terms of the safety of the patients and the clinical management?	(1) No improvement (2) Slight improvement (3) Significant improvement (4) Great improvement
How much do you think the use of electronic medical devices can help the patient care pathway?	(1) Little (2) Fairly (3) Very much

**Table 2:** LIMS study survey results.

What is your age?	20–30 yo: 12.5% 31–40 yo: 31.3% 41–50 yo: 37.5% 50+: 18.7%
How many years have you been working in the healthcare field?	<5: 6% 5–10: 12.5% 11–15: 19% 16–20: 43% 20+: 19.5%
Does the use of wireless monitoring device allow you to be more effective and efficient in the care of patients?	Great: 68.8% Fair: 25% Little: 6.2%
Has it been able to establish a bond with the patient trough digital visits as well?	Definitely better than traditional visit: 62% Slightly better: 26% No difference: 12% Less: 0%
In your opinion, is the device easy to use?	It’s very intuitive and easy to use: 30.5% After a short practice the devices is easy to use: 69.5% It’s very difficult to use: 0%
Have you noticed a significant reduction in the time taken to detect vital parameters?	Significant reduction: 39% Slight reduction: 42% Minimal reduction: 19% No reduction: 0%
Since the start of the project, have you noticed any improvement regarding patient safety and clinical management?	Great: 62% Significant: 26% Slight: 12% No improvement: 0%
How much do you think the use of electronic medical devices can help the patient care pathway?	Very much: 62% Fairly: 38% Little: 0% No help: 0%

**Table 3:** SWOT analysis of main advantages and disadvantages of the wireless monitoring system experienced by the healthcare personnel.

Strengths	Weaknesses
Easy-to-use tools for patients and operators. Small, non invasive, and safe tools.	Absence of a secure and widespread internet network in every part of the national territory. Few devices in hospitals, both public and private, and in low-intensity care settings for elderly, frail patients and/or patients with numerous comorbidities. Absence of national standard for all devices and platform.
**Opportunities**	**Threats**
Ability to easily diagnose adverse events of therapies or subclinical disease states. Reduction in emergency room visits and hospitalizations. Reduction in hospitalization costs. Increased adherence to therapy, and involvement in the care process for patients and caregivers.	Patients and data privacy can be subject to privacy violation/abusive data access. Absence of a secure and widespread internet network in every part of the national territory can generate inequality of care.

## Data Availability

Data sharing is not applicable.
